# Novel Strategies in the Development of New Therapies, Drug Substances, and Drug Carriers Volume II

**DOI:** 10.3390/ijms24065621

**Published:** 2023-03-15

**Authors:** Andrzej Kutner, Geoffrey Brown, Enikö Kallay

**Affiliations:** 1 Department of Drug Chemistry, Faculty of Pharmacy, Medical University of Warsaw, 1 Banacha, 02-097 Warsaw, Poland; 2 School of Biomedical Sciences, Institute of Clinical Sciences, College of Medical and Dental Sciences, University of Birmingham, Birmingham B15 2TT, UK; 3 Department of Pathophysiology and Allergy Research, Center of Pathophysiology, Infectiology & Immunology, Medical University of Vienna, Währinger Gürtel 18-20, A-1090 Vienna, Austria

The highly successful previous Volume 1.0 of the Special Issue “Novel Strategies in the Development of New Therapies, Drug Substances, and Drug Carriers” [1], in the *International Journal of Molecular Sciences* (IJMS), edited also as a book [2], comprised 21 papers. We, therefore, reopened this topic as Volume 2.0 of the Special Issue in *IJMS.* The interest remained high, leading to the publication of 24 original research articles and reviews. As for Volume I, this Special Issue was also conceived to cover a wide range of processes of drug development at the molecular level, complying with the scope of the journal.

Despite several decades of intensive research and the development of several generations of increasingly selective and effective drugs, cancer is still the leading cause of death. For this reason, anticancer therapy, specifically against leukemia and lymphoma, and solid tumors, including prostate, melanoma, ovarian, lung, urinary bladder, and breast cancers, remains a consistently leading area of interest for researchers worldwide. The interest was shared also by the participants of the Interdisciplinary Conference on Drug Sciences, ACCORD 2022, held at the Medical University of Warsaw. Most of the papers published in this Issue were presented, at least in part, at this Conference, as invited lectures, scientific communications, and posters. The main subjects of the communications were manifold: markers in neurodegenerative disorders, mediators of inflammation, non-coding micro-RNAs, enzymes regulating cell division, measures to mitigate post-operative side effects of cancer treatment, genetic disorders such as cystic fibrosis, skin disorders, multifunctional biomaterials, plant-derived topical preparations, crystal forms of active pharmaceutical ingredients, innovative drug product formulations, quality control of drug substances and drug products, including the chemical stability of drug substances and impurity profile, drug membrane permeability and identification of drug candidates by virtual screening a compound library. 

Most chemotherapeutics are effective against the bulk of cancer cells but usually leave cancer stem cells intact. Therapeutics that target cancer stem cells may also provide a cure for cancer. Brown [3] reviewed the two rationales for targeting the retinoic acid receptor γ (RARγ), which is expressed selectively within primitive cells. RARγ is a putative oncogene for several cancers, and prostate cancer cells depend on RARγ for their survival. Antagonizing all RAR-caused necroptosis of prostate cancer stem cell-like cells, and antagonizing RARγ was sufficient to drive necroptosis. The normal prostate epithelium was less sensitive to the RARγ antagonist and pan-RAR antagonist than prostate cancer cells, while fibroblasts and blood mononuclear cells were insensitive. The author concluded that the RARγ-selective antagonist and pan-RAR antagonist are potential therapeutics in cancer treatment.

Bexarotene, a drug that is active against cutaneous T-cell lymphoma, interferes with retinoid X-receptor (RXR)-dependent pathways and might cause serious side effects such as hypothyroidism. Therefore, Jurutka et al. [4] synthesized analogs of this drug, aiming to retain its key function and avoid harmful side effects. The bexarotene alicyclic ring, aliphatic linker, and benzoic acid moiety were substituted with an isochroman ring and nitrogen heterocycles, respectively. The ability of the new analogs to agonize RXR compared to bexarotene was evaluated. Analogs were modeled for RXR binding affinity, and EC_50_ and IC_50_ values were determined for a leukemia cell line. The analogs were tested for their ability to activate liver-X receptor (LXR) and increase the level of triglycerides, as well as LXRE-mediated transcription of brain ApoE expression as a marker of neurodegenerative disorders. While some RXR agonists cross-signaled the retinoic acid receptor (RAR), interestingly, many of the new analogs presented in this paper showed reduced activity against RAR. The authors demonstrated that selective modifications of rexinoids, such as bexarotene, can lead to analogs with increased RXR selectivity, decreased cross-signaling, and improved anti-proliferative characteristics against a leukemia cell line.

To evaluate the correlation between the synergy of an MDM2 inhibitor (siremadlin) combined with a MEK inhibitor (trametinib) in vitro and in vivo, Witkowski et al. [5] evaluated the interaction between these anticancer agents at the level of pharmacokinetics (PK) and pharmacodynamics (PD). The authors examined the cytotoxicity of a siremadlin and trametinib combination against melanoma A375 cells. Calculated drug interaction showed high synergy between siremadlin and trametinib and an increase in the potency of the drug combination. The authors recommended coupling physiologically based PK/PD (PBPK/PD) modeling, with further studies using cancer xenograft models to select the optimal PD interaction parameter to translate in vitro synergy into an animal model.

To identify highly effective therapeutic strategies, Witkowski et al. [6] developed in vitro/in vivo translational methods for synergistic drug combinations. The authors modeled PBPK/PD for siremadlin, trametinib, and a combination of these agents at various dose levels and dosing schedules for an A375 melanoma cell xenograft mouse model. The modeling was based on in vitro absorption, distribution, metabolism, and excretion, and in vivo, PK/PD reported data or estimated by the Simcyp Animal simulator (V21). The developed PBPK/PD models revealed the interactions between siremadlin and trametinib at the PK and PD levels. The interaction at the PK level is presented by an interplay between absorption and tumor disposition levels, whereas the PD interaction is based entirely on the vitro data. From these studies, and based on in vitro and in vivo extrapolation, the synergistic and most efficacious dose levels and dosing schedules for combinations of siremadlin and trametinib in mice were effectively developed. 

To extend their previous studies that aimed to provide an optimal therapeutic scheme, Witkowski et al. [7] elaborated on PBPK/PD modeling and clinical trial simulation for siremadlin and trametinib combination for use in melanoma patients. Clinical information was obtained from reported data or the Simcyp simulator. The PBPK/PD models accounted for the interactions between siremadlin and trametinib at the PK and PD levels. PK characteristics were predicted based on animal studies, while PD was based on in vitro cytotoxicity. These data, combined with virtual clinical trials, allowed for the estimation of PK/PD profiles and identified melanoma patients for whom this therapy might be non-inferior to the standard dabrafenib and trametinib combination. The authors demonstrated that PBPK/PD modeling, combined with clinical trial simulation, allows for the design of clinical trials and for predicting the clinical effectiveness of anticancer drug combinations. 

Ibuprofen, an aryl propionic acid, is used to treat rheumatoid arthritis and is an over-the-counter drug for use to treat minor pains. Chemically, it is a racemate, and only (*S*)-ibuprofen shows therapeutic function. The chiral metabolic inversion may cause the accumulation of one of the enantiomers, leading to toxicity. To overcome the side effects of dextrorotary ibuprofen (DXI), Thiruchenthooran et al. [8] designed nanostructured lipid carriers (NLCs) of DXI to be used in anticancer therapy. The formulation was optimized by a two-level factorial design. The spherical shape of the NLCs prolonged DXI release. Moreover, DXI-NLCs were more stable than the parent drug. DXI-NLCs showed in vitro cytotoxicity and anticancer potential against breast cancer cells. Therefore, the authors suggested that DXI-NLCs might become a promising antiproliferative therapy that is especially effective against breast cancer.

Platinum (II) complexes are still used to treat almost half of all cancer patients, and they exhibit anticancer activity by interacting with DNA and inducing programmed cell death. They undergo several bio-transformations and can form reactive transient species, which can complex macromolecules such as DNA. Szefler et al. [9] investigated the interactions of oxaliplatin with vitamin B, as compared to native purines. The authors carried out quantum-chemical simulations, with the set representing atomic orbitals of the platinum atom, using the Polarizable Continuum Model in a water environment. Additionally, time-dependent density functional theory (TD-DFT) was employed to study molecular properties in the electronically excited state. Interactions of vitamin B and oxaliplatin were investigated using UV-VIS spectroscopy. The free-energy values indicated spontaneous reactions with mono-aqua and the preferred di-aqua derivatives of oxaliplatin. The free energy values obtained for vitamin B indicated a lower affinity of oxaliplatin compared with the respective values obtained for guanine, adenine, and cytosine. The exception is the mono-aqua form of vitamin B1 (thiamine) at the MN15/def2-TZVP levels of calculations. An application of atoms in molecules (AIM) theory revealed non-covalent interactions that were present in the studied complexes. The data computed by the authors showed a good agreement with the experimental spectroscopic properties of the complexes. 

By interacting with nucleobases of DNA, platinum complexes form mono- and di-aqua products. These products are further complexed with guanine or adenine, leading to the death of cancer cells. The structures of vitamin B resemble the structures of nucleobases. Therefore, Szefler and Czeleń [10] reviewed theoretical and experimental studies of the interactions of vitamins B with Pt (II) complexes, as compared to guanine. Two levels of simulations were implemented at the theoretical level as well as the polarizable continuum model in an aqueous environment. Free-energy values showed spontaneous reactions with mono- and di-aqua derivatives of cisplatin and oxaliplatin. However, interactions with di-aqua derivatives are preferred. The strength of these interactions was also compared. Carboplatin products had the weakest interaction with the studied structures. The authors demonstrated the presence of non-covalent interactions in the complexes. Contrary to expectations, vitamin B formed weaker complexes with the products of hydrolysis of chemotherapeutics compared to nucleobases.

Overexpression of the enzymes that regulate cell division, such as cyclin-dependent kinases (CDK), is a key factor that contributes to carcinogenesis. Czeleń et al. [11] selected an indole derivative, isatin, as a freely available starting point for developing inhibitors for CDKs. Based on computational chemistry, docking, and molecular dynamics, the authors designed a series of potential inhibitors of the CDK2 enzyme, using isatin and benzoyl hydrazine as structure templates to synthesize newly selected analogs. The physicochemical properties of the analogs were well characterized. The authors successfully evaluated some new analogs as potential inhibitors of the CDK2 enzyme.

Metabolic resistance determines the lifetime and, therefore, the applicability of a compound as a drug substance candidate. For this reason, Żołek et al. [12] performed rigid docking supported by molecular dynamics simulation using the known structure of the native CYP3A4 enzyme to predict the metabolic resistance of analogs of 1,25-dihydroxyvitamin D_2_ (1,25D2) in vivo. The microsomal cytochrome P450 3A4 (CYP3A4) and mitochondrial cytochrome P450 24A1 (CYP24A1) hydroxylating enzymes both metabolize vitamin D and its analogs. The three-dimensional (3D) structure of the full-length native human CYP3A4 was solved, but the respective structure of the main vitamin D hydroxylating CYP24A1 enzyme remains unknown. Modeling using 3D data from human CYP3A4 provided a rationale for the substantial differences in the metabolic conversion of the side-chain geometric analogs of 1,25D2. The calculated free enthalpy of the binding of an analog of 1,25D2 to CYP3A4 agreed with the experimentally observed conversion of the analog by CYP24A1. This way, the authors proved that the metabolic resistance of an analog of 1,25D2 against the main vitamin D hydroxylating enzyme CYP24A1 can be predicted and explained by the binding strength of the analog to the known 3D structure of the CYP3A4 enzyme.

Invasive breast cancer therapy requires the development and selection of appropriate measures to mitigate post-operative side effects. Pawlicka et al. [13] evaluated the safety and potency of genistein (GE) using breast cancer MCF-7 cells and BJ skin fibroblasts. In a concentration-dependent manner, genistein affected both healthy dermal BJ fibroblasts and MCF-7 cells. Genistein at lower concentrations of 10 and 20 μM increased the abundance of dermal fibroblasts. However, genistein at a higher concentration, above 50 μM, was detrimental to fibroblasts at longer exposure times. Genistein shows high potential regarding the treatment of skin injuries, wounds, and surgical scars in women during and after breast cancer treatment. Even so, the authors recommended the need to be very cautious in selecting a concentration of isoflavonoids for use in treatment.

Given that isoflavonoids, such as genistein (GE), are effective antioxidants with antitumor activity, Stolarczyk et al. [14] characterized oxidation products from structurally related thiogenistein (TGE), both in solution and on the 2D surface of the Au electrode as a self-assembling TGE monolayer, using electrospray ionization mass spectrometry and Fourier-transform infrared spectroscopy. Density functional theory was used to support the experimental results for the estimation of the antioxidant activity of TGE, as well as for the molecular modeling of oxidation products. TGE showed high cytotoxic activity against human breast cancer cells and neutralized the production of lipopolysaccharide (LPS)-induced reactive oxygen species (ROS) more efficiently than GE. TGE also exhibited (2,2′-azino-bis-3-(ethylbenzothiazoline-6-sulphonic acid (ABTS) radical scavenging ability. TGE redox properties were related to its pharmacological activities. Most importantly, the authors demonstrated that the cytotoxic activity of TGE against human breast cancer cells was almost twice as high as that of GE. 

Human neutrophil elastase (HNE), a serine protease of the chymotrypsin family, can hydrolyze extracellular matrix proteins. Therefore, HNE is a major mediator of inflammation and has become a therapeutic target for small-molecule inhibitors. Donarska et al. [15] designed and synthesized a series of thiazoles, based on 3,3-diethylazetidine-2,4-dione, as new HNE inhibitors in the nanomolar range. The molecular docking study revealed a good correlation between the binding energies, indicating that the inhibition was largely dependent on the ligand alignment in the binding cavity. Most of the active compounds were stable and had antiproliferative activity against human leukemia, lung carcinoma, breast adenocarcinoma, and urinary bladder carcinoma cells, with IC_50_ values in the micromolar range. Additionally, some of the compounds induced growth arrest at the G2/M phase of the cell cycle and apoptosis via caspase-3 activation. The authors suggested that these new compounds might be effective against cancer and other diseases in which immunoreactive HNE is released.

Non-coding micro-RNAs (miRNAs) play an important role in the response of cancer cells to drug treatment by regulating the protein expression responsible for cell growth and proliferation. Gleba et al. [16] investigated the correlation between selected miRNAs and the proteins that they regulate in response to the active endogenous forms of vitamin D_3_, calcitriol, and tacalcitol by using several leukemia and lymphoma cell lines. Five miRNAs were selected as well as the respective regulated proteins. The authors concluded that the level of selected miRNAs correlated well with the levels of the proteins. They also identified some miRNAs that were most likely responsible for the anticancer activity of the most active forms of vitamin D_3_ in human leukemias and lymphomas. 

4,6,4′-Trimethylangelicin (TMA) is a promising agent for the treatment of cystic fibrosis, a genetic disorder that is caused by mutations in the gene for transmembrane conductance regulator (CFTR) protein. However, TMA shows serious disadvantages, such as poor solubility, phototoxicity, and mutagenicity. To overcome these side effects, Vaccarin et al. [17] designed and synthesized a library of TMA analogs. The authors showed that bulky aromatic substituents at C-4 of TMA impaired DNA intercalation and prevented the photoreaction of the lactone or furan double bond of the furocoumarin scaffold with the nucleic acid. Based on this finding, the authors obtained 4-phenyl- and 6-phenyl- analogs showing an F508del CFTR rescue ability. Interdisciplinary studies confirmed that the analogs lacked side effects. Pharmacokinetic studies revealed a favorable profile, especially after the incorporation of the analogs into lipid formulations. The authors concluded that the analogs are good candidates for novel CFTR correctors based on the angelicin scaffold. 

The increasing number of orthopaedical surgeries and the need to replace bone tissue have stimulated interest in developing multifunctional biomaterials to be used in bone diseases. Hence, Pajor et al. [18] searched for synthetic materials as carriers for delivering drugs, such as antibiotics, to combat surgical site infections. The authors studied the physicochemical properties and biological activity of different types of porous granules containing silver or gallium ions. Hydroxyapatite powders were doped with Ga^3+^ or Ag^+^. Then, the powders were used to fabricate ceramic micro granules and alginate/hydroxyapatite composite granules (AgT and GaT). The AgT and GaT granules showed high porosity and a large specific surface, whereas the micro granules contained very fine and numerous micropores. As expected, the granules released the incorporated ions slowly. All the granules except AgT were found to be non-cytotoxic. The authors subjected the granules to various antibacterial tests against key bacterial strains, which revealed that the new material had high antibacterial potency.

Skin disorders of different etiology, such as dermatitis, atopic dermatitis, eczema, psoriasis, wounds, burns, and others, are widespread in the population. For this reason, Melnyk et al. [19] first reviewed data on the plant-derived topical preparations used in Poland and Ukraine and then indicated future studies based on a recent understanding of the etiology of skin diseases. In severe cases, skin diseases require the topical application of antibiotics, steroids, and calcineurin inhibitors. Milder symptoms are treated with other medications, dietary supplements, and cosmetic products of plant origin. These skin care products were applied in various pharmaceutical formulations, including raw infusions, tinctures, creams, and ointments. The mechanisms of the beneficial effects of these treatments are often unclear. Recent developments in the role of the skin microbiota in maintaining skin homeostasis have helped researchers to understand the function of topically applied products of plant origin. As the authors concluded, knowledge of the medications’ interaction with the skin microbial ecosystem network remains to be upgraded. 

The existence of multiple crystal forms of an active pharmaceutical ingredient (API) is a serious challenge to the selection of the most suitable solid for drug development. It is also important for intellectual property protection. Braga et al. [20] reviewed the scientific, commercial, and ethical importance of investigating the crystalline forms (polymorphs, hydrates, and co-crystals) of active pharmaceutical ingredients. The physicochemical properties, such as solubility, dissolution rate, thermal stability, processability, therapeutic efficacy, etc., of the solid active pharmaceutical ingredient, usually depend on the crystal form and influence the efficacy of the drug product. The authors suggest that in addition to improving the understanding and control of the crystal form of the API, the study of hydrates and co-crystals may afford the alternative, often improved, innovative properties of the drug. The author provided examples of handling multiple crystal forms and showed how to exploit the intentional preparation of new crystalline forms as innovation. 

The quality control of drug products and the appropriate physicochemical properties of drug substances, including chemical stability and the right impurity profile, were of interest to several contributors. Since the worldwide crisis of contamination of medicinal products with nitrosamine, effective trace-level analysis of these contaminants is of continuing importance to the quality control of medicines. Witkowska et al. [21] developed a novel GC-MS method with electron ionization and microextraction for the simultaneous determination of main carcinogenic nitrosamines in a range of representative active pharmaceutical ingredients. The method demonstrated good linearity and wide limits of detection. The trace-level GC-MS method was specific, accurate, and precise. Nitrosamines were not detected in most of the APIs screened. However, in some of them, one nitrosamine was detected at the limit level of quantitation. Therefore, the authors stated that the novel method can be effectively applied for tracing nitrosamines in a range of APIs and can be used for the routine quality control of APIs to ensure the safety and high quality of medicinal products.

The chemical stability of drug substances is one of the major challenges in drug development. In this respect, Żyżyńska-Granica et al. [22] evaluated the stability of two biologically active peptide drug candidates that combine both opioid and neurotensin pharmacophores in one hybrid compound. The hybrids are structurally similar and differ only in an amino acid at position 9 of the peptide chain. Isoleucine in the parent compound was replaced with its isomer, *tert*-leucine. The study aimed to assess and compare the chemical stability of the hybrids. Contrary to the change in biological activity, substituting tert-leucine with isoleucine did not affect chemical stability. Neither alkaline nor acidic hydrolysis and oxidative degradation resulted in stability differences between the hybrids. However, the number of degradation products under acidic conditions increased. In summary, the authors showed that the introduced modification reduced the compound’s half-life.

Gurba-Bryśkiewicz et al. [23] carried out a complete Analytical Quality by Design (AQbD) approach, including screening, optimization, and validation, for the development of a new method for the quantitative determination of the complete impurity profile of an innovative pharmaceutical substance with structure-based pre-development. The authors demonstrated a novel approach to the development of an ultra HPLC method using the AQbD with the Design of an Experiment (DOE). The method was applied for the quantitative determination of the impurity profile of a JAK/ROCK inhibitor (CPL409116) during preclinical drug discovery. The critical method parameters (CMPs), including the stationary phase of the columns, pH of the aqueous mobile phase, and composition of the organic mobile phase, were tested extensively. The authors selected the resolution between the peaks and peak symmetry of analytes as the critical method attributes (CMAs). The influence of various levels of CMPs on the CMAs was evaluated based on a full fractional design. The robustness tests were established from the knowledge space of the screening and completed by fractional factorial design. Method-operable design region (MODR) was created. Monte-Carlo simulations provided the probability of complying with the specifications. The authors documented that the optimized method is specific, linear, precise, and robust. 

In the field of innovative agents used for biomedical applications, glutaraldehyde is one of the most popular synthetic cross-linkers for drug product formulations. However, the unreacted cross-linker can be released from the drug product and cause unexpected side effects. To overcome this difficulty, Wegrzynowska-Drzymalska et al. [24] obtained dialdehyde starch nanocrystals (NDASs) as an alternative to the commonly used cross-linking agents. Then, NDASs were used for chemical cross-linking of native chitosan (CS), gelatin (Gel), and a mixture of these two biopolymers (CS-Gel). The thin films of the materials were characterized by Attenuated Total Reflectance FTIR, Scanning Electron Microscopy (SEM), and X-ray diffraction (XRD), and toxicity was tested by the Microtox test. Thermal and mechanical properties were determined by thermal gravimetric analysis (TGA) and tensile testing. Moreover, all cross-linked biopolymers were characterized by hydrophilic character, swelling ability, and protein absorption. The authors revealed that dialdehyde starch nanocrystals form beneficial plant-derived cross-linking agents, leading to cross-linked biopolymers that are advantageous to biomedical applications.

Membrane permeability is important in the development of therapeutic peptides of large molecular size. Therefore, Mazzanti et al. [25] computed the permeability coefficient of a cyclic decapeptide by comparing two physical models. The authors applied the inhomogeneous solubility–diffusion model (ISDM) requiring umbrella–sampling simulations. A chemical kinetics model was also used including multiple unconstrained simulations (MSM). The authors compared the accuracy of the approaches and concluded that the MSM method requires much more computer resources than the ISDM method while giving comparable predictions of the membrane permeability of peptides.

Papain-like protease (PL^pro^) is a significant target for anti-COVID-19 drugs. Therefore, Wu et al. [26] virtually screened a large library of compounds against this protein and identified several drug candidates. The binding energy of the candidates was estimated as higher than proposed in previous studies. By analyzing the docking results, the authors demonstrated that the critical interactions between the candidates and PL^pro^ revealed by the computations were consistent with the biological experiments. The predicted binding energies among selected candidates and absorption, distribution, metabolism, and excretion (ADME) characteristics were consistent with their IC_50_ values.

The papers within this Special Issue cover a wide range of topics regarding the development of new drug substances, new drug carriers, and drug products. As conceived, the topics in the Issue cover the identification of the molecular target of new drugs, docking supported by molecular dynamics simulation, quantum-chemical simulations, studies of protein–drug interactions, modeling and optimization of the structure of small molecules with drug-like activity, determination of the functional profile, pre-formulation studies, new pharmaceutical formulations, preclinical development to the design, and prediction of the efficacy of agents in clinical trials. Elucidation of the molecular mechanism of action is now an integral prerequisite for transforming an active substance into a drug candidate.

## References

[1] Kutner A., Brown G., Kallay E. International Journal of Molecular Sciences, Special Issue “Novel Strategies in the Development of New Therapies, Drug Substances, and Drug Carriers”. https://www.mdpi.com/journal/ijms/special_issues/ACCORD2021.

[2] Kutner A., Brown G., Kallay E. (2022). Novel Strategies in the Development of New Therapies, Drug Substances, and Drug Carriers.

[3] Brown G. (2022). Antagonizing RARγ Drives Necroptosis of Cancer Stem Cells. Int. J. Mol. Sci..

[4] Jurutka P.W., di Martino O., Reshi S., Mallick S., Sausedo M.A., Moen G.A., Lee I.J., Ivan D.J., Krall T.D., Peoples S.J. (2022). An Isochroman Analog of CD3254 and Allyl-, Isochroman-Analogs of NEt-TMN Prove to Be More Potent Retinoid-X-Receptor (RXR) Selective Agonists Than Bexarotene. Int. J. Mol. Sci..

[5] Witkowski J., Polak S., Rogulski Z., Pawelec D. (2022). In Vitro/In Vivo Translation of Synergistic Combination of MDM2 and MEK Inhibitors in Melanoma Using PBPK/PD Modelling: Part I. Int. J. Mol. Sci..

[6] Witkowski J., Polak S., Rogulski Z., Pawelec D. (2022). In Vitro/In Vivo Translation of Synergistic Combination of MDM2 and MEK Inhibitors in Melanoma Using PBPK/PD Modelling: Part II. Int. J. Mol. Sci..

[7] Witkowski J., Polak S., Pawelec D., Rogulski Z. (2023). In Vitro/In Vivo Translation of Synergistic Combination of MDM2 and MEK Inhibitors in Melanoma Using PBPK/PD Modelling: Part III. Int. J. Mol. Sci..

[8] Thiruchenthooran V., Świtalska M., Bonilla L., Espina M., García M.L., Wietrzyk J., Sánchez-López E., Gliszczyńska A. (2022). Novel Strategies against Cancer: Dexibuprofen-Loaded Nanostructured Lipid Carriers. Int. J. Mol. Sci..

[9] Szefler B., Czeleń P., Wojtkowiak K., Jezierska A. (2022). Affinities to Oxaliplatin: Vitamins from B Group vs. Nucleobases. Int. J. Mol. Sci..

[10] Szefler B., Czeleń P. (2023). Will the Interactions of Some Platinum (II)-Based Drugs with B-Vitamins Reduce Their Therapeutic Effect on Cancer Patients? Comparison of Chemotherapeutic Agents such as Cisplatin, Carboplatin, and Oxaliplatin—A Review. Int. J. Mol. Sci..

[11] Czeleń P., Skotnicka A., Szefler B. (2022). Designing and Synthesis of New Isatin Derivatives as Potential CDK2 Inhibitors. Int. J. Mol. Sci..

[12] Żołek T., Yasuda K., Brown G., Sakaki T., Kutner A. (2022). In Silico Prediction of the Metabolic Resistance of Vitamin D Analogs against CYP3A4 Metabolizing Enzyme. Int. J. Mol. Sci..

[13] Pawlicka M.A., Zmorzyński S., Popek-Marciniec S., Filip A.A. (2022). The Effects of Genistein at Different Concentrations on *MCF-7* Breast Cancer Cells and *BJ* Dermal Fibroblasts. Int. J. Mol. Sci..

[14] Stolarczyk E.U., Strzempek W., Łaszcz M., Leś A., Menaszek E., Stolarczyk K. (2022). Thiogenistein—Antioxidant Chemistry, Antitumor Activity, and Structure Elucidation of New Oxidation Products. Int. J. Mol. Sci..

[15] Donarska B., Świtalska M., Wietrzyk J., Płaziński W., Mizerska-Kowalska M., Zdzisińska B., Łączkowski K.Z. (2022). Discovery of New 3,3-Diethylazetidine-2,4-dione Based Thiazoles as Nanomolar Human Neutrophil Elastase Inhibitors with Broad-Spectrum Antiproliferative Activity. Int. J. Mol. Sci..

[16] Gleba J.J., Kłopotowska D., Banach J., Mielko K.A., Turlej E., Maciejewska M., Kutner A., Wietrzyk J. (2022). Micro-RNAs in Response to Active Forms of Vitamin D_3_ in Human Leukemia and Lymphoma Cells. Int. J. Mol. Sci..

[17] Vaccarin C., Gabbia D., Franceschinis E., De Martin S., Roverso M., Bogialli S., Sacchetti G., Tupini C., Lampronti I., Gambari R. (2022). Improved Trimethylangelicin Analogs for Cystic Fibrosis: Design, Synthesis and Preliminary Screening. Int. J. Mol. Sci..

[18] Pajor K., Michalicha A., Belcarz A., Pajchel L., Zgadzaj A., Wojas F., Kolmas J. (2022). Antibacterial and Cytotoxicity Evaluation of New Hydroxyapatite-Based Granules Containing Silver or Gallium Ions with Potential Use as Bone Substitutes. Int. J. Mol. Sci..

[19] Melnyk N., Vlasova I., Skowrońska W., Bazylko A., Piwowarski J.P., Granica S. (2022). Current Knowledge on Interactions of Plant Materials Traditionally Used in Skin Diseases in Poland and Ukraine with Human Skin Microbiota. Int. J. Mol. Sci..

[20] Braga D., Casali L., Grepioni F. (2022). The Relevance of Crystal Forms in the Pharmaceutical Field: Sword of Damocles or Innovation Tools?. Int. J. Mol. Sci..

[21] Witkowska A.B., Giebułtowicz J., Dąbrowska M., Stolarczyk E.U. (2022). Development of a Sensitive Screening Method for Simultaneous Determination of Nine Genotoxic Nitrosamines in Active Pharmaceutical Ingredients by GC-MS. Int. J. Mol. Sci..

[22] Żyżyńska-Granica B., Mollica A., Stefanucci A., Granica S., Kleczkowska P. (2022). Comparative Study of Chemical Stability of a PK20 Opioid–Neurotensin Hybrid Peptide and Its Analogue [Ile^9^]PK20—The Effect of Isomerism of a Single Amino Acid. Int. J. Mol. Sci..

[23] Gurba-Bryśkiewicz L., Dawid U., Smuga D.A., Maruszak W., Delis M., Szymczak K., Stypik B., Moroz A., Błocka A., Mroczkiewicz M. (2022). Implementation of QbD Approach to the Development of Chromatographic Methods for the Determination of Complete Impurity Profile of Substance on the Preclinical and Clinical Step of Drug Discovery Studies. Int. J. Mol. Sci..

[24] Wegrzynowska-Drzymalska K., Mylkie K., Nowak P., Mlynarczyk D.T., Chelminiak-Dudkiewicz D., Kaczmarek H., Goslinski T., Ziegler-Borowska M. (2022). Dialdehyde Starch Nanocrystals as a Novel Cross-Linker for Biomaterials Able to Interact with Human Serum Proteins. Int. J. Mol. Sci..

[25] Mazzanti L., Ha-Duong T. (2023). Understanding Passive Membrane Permeation of Peptides: Physical Models and Sampling Methods Compared. Int. J. Mol. Sci..

[26] Wu Y., Pegan S.D., Crich D., Lou L., Nicole Mullininx L., Starling E.B., Booth C., Chishom A.E., Chang K.Y., Xie Z.-R. (2023). Identifying Drug Candidates for COVID-19 with Large-Scale Drug Screening. Int. J. Mol. Sci..

